# Neural Processes of Proactive and Reactive Controls Modulated by Motor-Skill Experiences

**DOI:** 10.3389/fnhum.2019.00404

**Published:** 2019-11-14

**Authors:** Qiuhua Yu, Bolton K. H. Chau, Bess Y. H. Lam, Alex W. K. Wong, Jiaxin Peng, Chetwyn C. H. Chan

**Affiliations:** ^1^Department of Rehabilitation Medicine, The First Affiliated Hospital, Sun Yat-sen University, Guangzhou, China; ^2^Applied Cognitive Neuroscience Laboratory, Department of Rehabilitation Sciences, The Hong Kong Polytechnic University, Hong Kong, China; ^3^Program in Occupational Therapy, Washington University School of Medicine, St. Louis, MO, United States; ^4^Department of Neurology, Washington University School of Medicine, St. Louis, MO, United States; ^5^Department of Education, Shaoguan University, Shaoguan, China; ^6^University Research Facility in Behavioral and Systems Neuroscience, The Hong Kong Polytechnic University, Hong Kong, China

**Keywords:** proactive control, reactive control, task switching, ERP, motor skills

## Abstract

This study investigated the experience of open and closed motor skills on modulating proactive and reactive control processes in task switching. Fifty-four participants who were open-skilled (*n* = 18) or closed-skilled athletes (*n* = 18) or non-athletic adults (*n* = 18) completed a cued task-switching paradigm task. This task tapped into proactive or reactive controls of executive functions under different validity conditions. Electroencephalograms of the participants were captured during the task. In the 100% validity condition, the open-skilled participants showed significantly lower switch cost of response time than the closed-skilled and control participants. Results showed that the open-skilled participants had less positive-going parietal cue-locked P3 in the switch than repeat trials. Participants in the control group showed more positive-going cue-locked P3 in the switch than repeat trials, whereas the closed-skilled participants had no significant differences between the two types of trials. In the 50% validity condition, the open- and closed-skilled participants had less switch cost of response time than the control participants. Participants in the open- and closed-skilled groups showed less positive-going parietal stimulus-locked P3 in the switch than repeat trials, which was not the case for those in the control group. Our findings confirm the dissociation between proactive and reactive controls in relation to their modulations by the different motor-skill experiences. Both proactive and reactive controls of executive functions could be strengthened by exposing individuals to anticipatory or non-anticipatory enriched environments, suggesting proactive and reactive controls involved in motor-skill development seem to be transferable to domain-general executive functions.

## Introduction

A high level of motor skills has been associated with improved executive functions. Sports are physical activities that require high levels of motor skills. Individuals who engaged in fencing (Chan et al., 2011), baseball (Kida et al., 2005), and soccer were reported to have better executive functions than the controls (Verburgh et al., 2014). Researchers have suggested that the enhancement in executive functions could be related to the neural plasticity brought about by the long-term aerobic fitness and cognitive trainings involved in these sports (Voss et al., 2010; Chan et al., 2011). Common features of the physical activities described above are playing facing opponents and in changing external environments, which demand open motor skills. Open motor skills involve generation of physical responses to dynamically and externally paced environment (Wang et al., 2013a; Yu et al., 2017). Contrary to open motor skills is closed motor skills, which requires participants to generate responses that are relatively consistent, stationary, and self-paced (Wang et al., 2013a; Yu et al., 2017). The typical physical activities involving closed motor skills are swimming, and track and field. In view of the differences between open and closed motor skills, it is intuitive that participants of “closed” physical activities would have gained lower level of executive functions than those of “open” physical activities. For instance, open-skilled participants were revealed to have higher levels of inhibitory control than the closed-skilled counterparts (Yamashiro et al., 2015). Nevertheless, in an earlier behavioral study, we reported that, by employing a dual cognitive control model for executive functions (Chevalier et al., 2015; Tarantino et al., 2016), both the open- and closed-skilled participants showed significantly higher levels of reactive control of executive functions than the controls (Yu et al., 2017). The differences between the two experimental groups are in the proactive versus reactive control. In this paper, we aimed to employ electroencephalogram to understand the neural mechanisms underlying how different types of motor skills would associate with proactive or reactive controls among participants engaging in physical activities.

Proactive control is an early selection process, which can optimally bias attention, perception, and action systems in a goal-driven manner (Braver, 2012). Information selected early in the process is to be deployed before the response-demanding event occurs. In contrast, reactive control is to resolve the interference imperatively after the response-demanding event appears (Braver, 2012). Physical activities dominated by open motor skills involve predictions of outcomes upon the actions of opponents and teammates for producing rapid and accurate responses (Jin et al., 2011; Abreu et al., 2012; Yu et al., 2016). These cognitive processes are comparable to those described in proactive control. In contrast, the physical activities dominated by closed motor skills less involve the prediction of actions of others before giving the responses. In addition, open motor skills require the participants to rapidly inhibit inappropriate actions, and switch from an intended movement to an appropriate one in an unpredictable environment (Taddei et al., 2012; Yu et al., 2017). The imperative inhibition and switching process are comparable to those described in reactive control. Tsai and Wang (2015) explored the effects of open- (e.g., badminton and table tennis) or closed-skilled (e.g., jogging and swimming) training in the reactive control of task switching for elderly subjects. Their results showed that open-skilled group had larger P3 amplitude in switch condition than closed-skilled and control groups, but had comparable P3 amplitude in repeat condition with the other two groups. All the studies revealed employed a unidimensional perspective of executive control. The results are that open-skilled groups had higher level of executive functions in reactive control associated with enhanced P3 than closed-skilled groups. No study has been conducted to explore and explain the possible gains in different time processes of executive functions differentially in the open- and closed-skilled groups. The dual cognitive control model of executive function offers a theoretical basis for addressing the potential differentiation in the cognitive gains due to the engagement in these physical activities.

Neural processes related to proactive and reactive controls can be examined by using a cued task-switching paradigm with electroencephalogram (EEG). The cued task-switching paradigm involves participants predicting a switch of task rule based on the information embedded in the cue and subsequently giving a response according to the new rule. The task cue can be fully predictive (100% validity) or fully non-predictive (50% validity), which makes possible elicitation of the event-related potentials (ERPs) for reflecting the proactive or reactive control processes, respectively. The common ERPs associated with proactive control in the task-switching paradigm reported are the parietally distributed P3 elicited by the cue (called cue-locked P3) (Gajewski and Falkenstein, 2011; Tarantino et al., 2016) and the frontocentrally distributed contingent negative variant (CNV) (Tarantino et al., 2016). In contrast, the frontally distributed N2 (Hsieh and Wu, 2011) and parietally distributed P3 (called stimulus-locked P3) (Scisco et al., 2008; Tarantino et al., 2016) elicited by the stimulus were reported to associate with the reactive control in task switching. The cue-locked P3 can be identified within the 300–600 ms time-window after the appearance of a predictive cue (West et al., 2012; Tarantino et al., 2016). This component was suggested to reflect task reconfiguration – anticipatory updating of task goals and/or action rules in working memory (Nicholson et al., 2006; Gajewski and Falkenstein, 2011). The CNV is a slow wave elicited prior to the onset of the target stimulus (Funderud et al., 2013). It reflects anticipatory attention and motor preparation for the upcoming target stimulus (Funderud et al., 2013; Grane et al., 2016). Thus, the amplitude of CNV could be modulated by the response-related parameters embedded in the task cue (Scheibe et al., 2009; Linssen et al., 2013), e.g., the cue validity. Previous studies also showed that Bereitschaftspotential (BP), comparable to CNV, was more negative-going in athletes than non-athletes (Bianco et al., 2017a, b). This finding suggested that the athletes would have better motor preparation than the non-athletes. However, Wang et al. (2013b) did not reveal significant differences in motor preparation between the open- and closed-skilled players.

The N2 component has been employed as a marker reflecting reactive control – suppression of conflict responses (Rushworth et al., 2002; Hsieh and Wu, 2011). More negative-going frontal N2 was shown to associate with the switching to a new response set. The stimulus-locked P3 can be identified after the appearance of a response-demanding stimulus (Swainson et al., 2006; Tarantino et al., 2016). In the predictive cue condition, the stimulus-locked P3 accounts for stimulus-response set implementation (Jamadar et al., 2010; Gajewski and Falkenstein, 2011; Tarantino et al., 2016) or task-specific evaluation of a target stimulus (Swainson et al., 2006). The amplitude difference of stimulus-locked P3 between switch and repeat trials was negatively related to the switch cost of the response time when the cue was predictive (Li et al., 2012), suggesting that stimulus-locked P3 in reactive control was related to the performance in task switching. In the non-predictive cue condition, stimulus-locked P3 is associated with updating of the task goal or task rules (Hillman et al., 2006; Scisco et al., 2008). It was more positive-going in the switch than repeat trials, because more attentional resources required for subsequent memory updating in reactive control (Hillman et al., 2006; Scisco et al., 2008). However, Kamijo and Takeda (2010) reported intensive experience of physical training, regardless of type of sport, showed less positive stimulus-locked P3 in the switch than repeat trials in an alternating-runs switching paradigm. The reason was likely that these studies showed differences in the task difficulties, which P3 component was sensitive to Kamijo and Takeda (2010).

In this study, a cued task-switching task was employed for eliciting the proactive and reactive control processes modulated by the participants with experience of open- or closed-skilled physical activities. As the participants had received intensive training of two types of motor skills, we hypothesized that in the trials with predictive cues (100% validity), the open-skilled participants would show fewer positive differences in the between-trial (switch verse repeat) cue-locked P3 compared with the closed-skilled participants due to employing more anticipation in the open-skilled training. It was also hypothesized that in the trials with non-predictive cues (50% validity), the open-skilled participants would show fewer positive differences in the between-trial stimulus-locked P3 than the closed-skilled and control ones due to the imperative switch in the unpredictable environment. More negative-going CNV in the open- and closed-skilled than controls participants were anticipated due to better preparation in the former two groups. Comparable N2 amplitudes were anticipated in the open- and closed-skilled participants. The performances in the neural processes of the 75% validity condition would be between those of the 100 and 50% validity conditions.

## Materials and Methods

### Participants

Fifty-four university students were recruited via convenience sampling. Among them, 18 (8 females and 10 males) were members of the university badminton team (open-skilled group), 18 (7 females and 11 males) were members of the university track and field team (closed-skilled group), and 18 (9 females and 9 males) declared they had not engaged in any professional or amateur sport (control group). The selection of badminton and track and field athletes as the open- and closed-skilled participants was made reference to those recruited in Wang and Tu (2017) and Wang et al. (2017). The results obtained would have more meaningful comparisons with those reported by Wang and Tu (2017) and Wang et al. (2017). Participants in each group had matched age and education levels (Table 1). The participants of the open- and closed-skilled groups had five or more years for professional motor skill practices. Each athlete had won prizes in open competitions and had no regular training in other sports. The levels of skill competences (in terms of winning international/local sport awards) were comparable between two groups (*n* = 2/16 for open-skilled versus *n* = 3/15 for closed-skilled). All of the participants were right-handed with normal or corrected-to-normal visual acuity, and had no history of neurological or cardiovascular disorders. Participants were not on regular medication. The participant’s cardiorespiratory fitness was assessed by the Queen’s College step test, which had been introduced in Yu et al.’s (2017). Ethical approval of this study was granted by the Departmental Research Committee of The Hong Kong Polytechnic University. Written informed consent was obtained from the participants before commencing the experiment for data collection.

**TABLE 1:** Demographic characteristics of the open-skilled, closed-skilled and control participants.

	**Open-skilled**	**Closed-skilled**	**Control**	***F*, *p***
Age, M (SD)	21.1 (2.2)	21.1 (2.0)	21.8 (2.1)	*F* = 0.77, *p* = 0.466
Weight kg, M (SD)	67.2 (11.6)	58.3 (9.4)	57.1 (10.2)	*F* = 5.00, *p* = 0.010
Height cm, M (SD)	170.3 (8.4)	169.9 (7.4)	165.1 (7.5)	*F* = 2.55, *p* = 0.088
BMI kg/m^2^, M (SD)	22.9 (2.4)	20.1 (2.0)	20.9 (2.6)	*F* = 7.06, *p* = 0.002
Years of professional motor skill practices^∗^, M (SD)	11.3 (2.7)	7.9 (1.6)	N.A.	*F* = 179.35, *p* < 0.001
Hours of professional motor skill practices each week, M (SD)	8.3 (1.8)	8.6 (1.6)	N.A.	*F* = 216.93 *p* < 0.001
VO2max mL^∗^kg^–1*^min^–1^, M (SD)	54.9 (9.3)	55.0 (10.2)	47.4 (10.4)	*F* = 3.42, *p* = 0.040
Other expertise outside of sports	Two participants engaged in playing musical instrument	Two participants engaged in playing musical instrument; one player in swimming	Two participants engaged in playing musical instrument; one player in wing chun	N.A.

*^∗^Denotes the regular motor skill practices under the coach guidance.*

### Experimental Task

**FIGURE 1:**
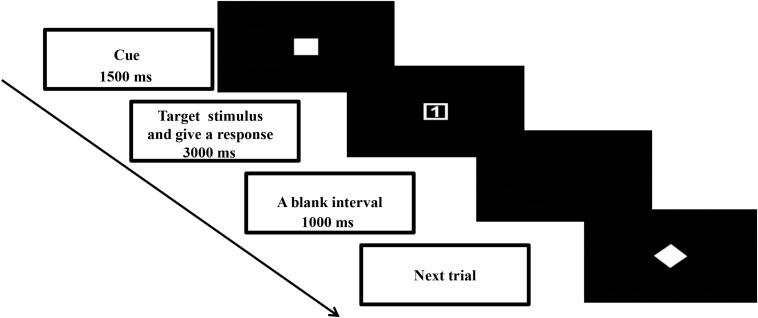
Schematic illustration of one typical trial in the cued task-switching paradigm.

This study used a cued task-switching paradigm to manipulate the proactive and reactive controls. Details of the task design were described in Yu et al. (2017). The time course of one typical trial is summarized in Figure 1. A trial began with presentation of a task cue (4 cm × 4 cm) at the center of the screen for 1500 ms. Then the task cue was replaced by a target stimulus (4 cm × 4 cm). Upon presentation of the target stimulus, the participant was asked to give a two-key sequential response correctly as soon as possible within 3000 ms. The next trial began once the response from the participant was registered. Next, a blank interval of 1000 ms appeared before the onset of the next trial.

In this paradigm, proactive and reactive controls were manipulated by means of cue validities. Three cue validities were used: 100, 75, and 50%. A 100% valid cue appeared as a solid square 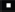 (or diamond 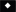), a 75% valid cue appeared as a hollow square 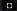 (or diamond 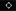), and a 50% valid cue appeared (as a solid star 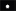). The participants were asked to prepare the response selection rules based on the cue (except for a 50% valid cue). A 100% valid cue meant the rule sets conveyed in the cue would be the same as those that appeared in the target stimulus. In this case, the participant could prepare to repeat the same task rule as the previous trial or switch to a new task rule based on the rule set conveyed in the cue. Thus, a 100% valid cue would elicit more proactive but fewer reactive control processes. A 50% valid cue meant that no information on the task sets in the subsequent response was provided. In such an ambivalent situation, the participant could not give a specific preparation for the response. The participant could repeat the same task rule or switch to a new task rule based on the target stimulus. It was expected that a 50% valid cue would elicit reactive rather than proactive control processes. A 75% valid cue was expected to elicit mental processes that were a combination of those in the 50 and 100% validity conditions. A digit (1 or 2) that appeared inside the shape (a square or diamond) formed the target stimulus (Figure 1). Two sets of response selection rules were used. Each rule involved two sets of two-key sequential responses delivered by the target stimulus with the same shape (square or diamond). For instance, one response selection rule was that a square with digit “1” was for the participant to press the “z” and then “n” keys on the keyboard, and a square with digit “2” was “x” and then “m”; the other response selection rule was that a diamond with digit “1” was “x” and then “n”, and a diamond with digit “2” was “z” and then “m.” The mappings between cue stimulus (square or diamond) and two response selection rules were counterbalanced across participants. Only the 75% validity condition had congruent and incongruent trials. Congruent trials featured the same hollow square or diamond shapes appearing in the cue and target. Incongruent trials were when the shapes displayed in the cue and target differed. Only valid congruent trials in the 75% validity condition were included in the data analyses. Each trial was defined as “repeat” or “switch” depending on the response selection rule. A repeat trial was that the response selection rule was the same as that in the previous trial; whereas that of a switch trial was different from the previous trial.

The ratio of the three types of cue validities was 1:1:1. There was same ratio of repeat to switch trials in each block. Trials were organized in counterbalanced orders and grouped into nine blocks, and each block had 140 trials. It took around 9 min to complete one block followed by a 4-to-5 min break. NeuroScan Stim2 software (NeuroScan, Inc., Sterling, VA, United States) was used for the fabrication of the trials. The time for completing the experimental task was approximately 1.5 h.

### Data Collection Procedures

#### Preparation

Each participant was asked to complete the demographic information sheet, which included years for professional motor skill practices, hours for professional motor skill practices per week, sports categories, and other expertise outside of sports (Table 1). Before engaging in the experimental task, each participant sat on a comfortable chair in front of a table inside a dimly lit and electrically isolated sound-proof chamber. A 15-inch computer monitor for showing trials was placed on the table at a distance of 65–75 cm. Each participant was required to first complete 100 practice trials. Standardized instructions and feedback were given to the participant throughout the training block. This was followed by a test block in which the participant completed 50 task trials and reached 90% accuracy before entering into the experiment. If the participant achieved an accuracy rate of less than 90%, he or she repeated the training block. The participant was also reminded to minimize eye blinks and to keep his or her eyes at the center of the monitor throughout the task.

#### Acquisition of ERP Data

Participants’ EEG signals were captured by a 64-channel Quik-cap equipped with 90 mm Ag/AgCl sintered electrodes, SynAmps2 Digital DC EEG amplifier, and Curry 7 software (NeuroScan, Inc., Sterling, VA, United States). Vertical and horizontal electrooculograms (EOGs) were captured with two pairs of electrodes placed on the supra- and infra-orbital areas of the left eye and the left and right orbital rims of both eyes, respectively. A ground electrode was positioned on the forehead in front of the Cz electrode. All the channels were referenced to the electrodes on the left and right mastoids. The EEG and EOG signals were sampled at a rate of 1000 Hz/channel. All EEG/EOG electrode impedances were set to below 5 kΩ. EEG signals were recorded from the beginning of each block of experimental tasks. The timing of all stimuli was recorded by Curry 7 software.

Offline signal preprocessing also employed Curry 7 software. EEG signals were digitally filtered with a band pass from 0.01 to 30 Hz. The covariance analysis algorithm was used when eye movement was detected. Then the EEG signals were segmented into the cue- and stimulus-locked epochs. Cue-locked epochs were defined as −200 ms before the cue to 1,500 ms after the cue, and stimulus-locked epochs were defined as −200 ms before the target to 1,000 ms after the target. Baseline corrections were referenced to the pre-stimulus interval. Epochs with an amplitude exceeding ± 80 μv and trials with incorrect responses were excluded from the subsequent averaging procedure. The cue- and stimulus-locked waveforms of each electrode were averaged separately for three cue validities (100, 75 versus 50%) and two task conditions (repeat versus switch). The number of epochs extracted for data analysis for each cue validity in each of the repeat or switch trials was around 140 for each group.

### Data Analysis

As the behavioral data of this study shared the same data set of a previous study conducted by the same research team, the detailed methods of analyzing the behavioral results of the participants can be found in Yu et al. (2017) and will not be repeated here. Analyses of the ERP data included the cue-locked P3, CNV, N2 and the stimulus-locked P3 elicited when participants engaged in the behavioral task. Independent component analysis (ICA) was conducted to confirm the time-windows set for extracting signals related to the cue-locked P3 (350–550 ms post-cue), CNV (1200–1500 ms post-cue), N2 (200–300 ms post-target), and stimulus-locked P3 (300–600 ms post-target). A short time-window was set for the cue-locked P3 for lowering the possible interferences to the CNV, as ICA results showed a slight overlap in the time-windows of these two components. In the analysis, the electrodes at the midline sites (Fz, Cz, and Pz) were included making reference to the results of previous studies that switch effects were maximal at the midline electrode sites (Gajewski and Falkenstein, 2011; Hsieh and Wu, 2011; Li et al., 2012). A four-way repeated measures ANCOVA for validity (100, 75 versus 50%) × trial (repeat versus switch) × site (Fz, Cz versus Pz) × group (open-skilled, closed-skilled versus control) was conducted to test the mean amplitudes of the cue-locked P3, CNV, N2, and stimulus-locked P3. The years of participants’ professional motor skill practices was the only covariate entered because of the significant between-group differences in this variable (Table 1). Another reason was that the years of professional motor skill practices rather than the MBI and VO2max was revealed to significantly predict the participants’ performances on the behavioral tasks in Yu et al. (2017). *Post hoc* pairwise comparisons with the Bonferroni adjustment were applied when significant main or interaction effects were observed. This study only included the amplitudes rather than latencies in the ERP data analyses because previous study reported no significant differences between groups and between trials on the P3 latency (Scisco et al., 2008). As the switch cost of response time was related to amplitudes of cue- or stimulus-locked P3 components (Jamadar et al., 2010; Li et al., 2012), the present study examined the relations among cue- and stimulus-locked P3 and behavioral performance (i.e., switch cost of response time) for different motor skills by using a hierarchical, stepwise regression analysis for each of the validity conditions. For each regression equation, two regressors were the mean amplitude differences between switch and repeat trials of cue- (cP3_S_-cP3_*R*_) and stimulus-locked P3 (sP3_S_-sP3_*R*_); two other regressors were the identities of the open- and closed-skilled groups (with the control group as the reference). The two-way interaction terms for the neural processes and group identities were cP3_S_-cP3_R_ × open-skilled; cP3_S_-cP3_R_ × closed-skilled; sP3_S_-sP3_R_ × open-skilled; and sP3_S_-sP3_R_ × closed-skilled. The variance inflation factor (VIF) ≥ 10 and Pearson’s correlation ≥ 0.85 were considered as indicators of strong multicollinearity between any of the two independent variables in a hierarchical regression model. The results showed no variable displayed strong multicollinearity, suggesting that none of the variables were related to each other. All analyses were performed with IBM SPSS statistics version 20.0 (IBM, Chicago, IL, United States).

## Results

**FIGURE 2:**
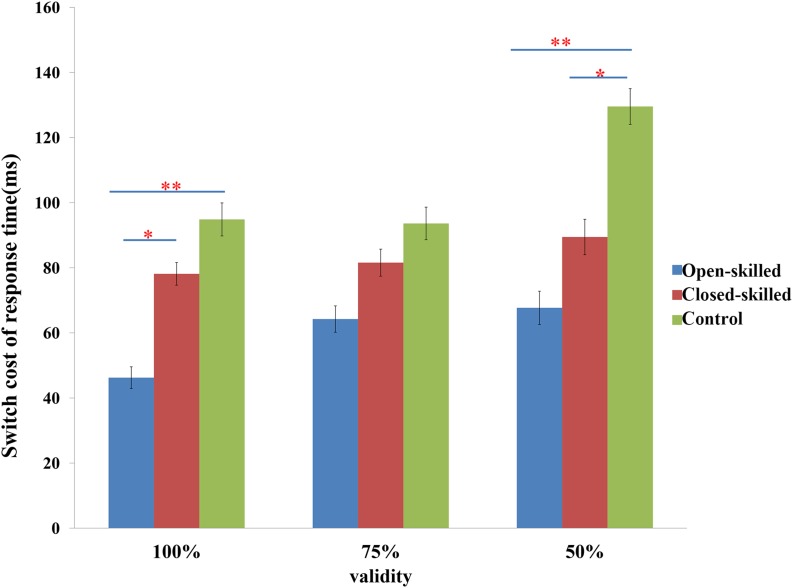
The switch cost of response times in task-switching paradigm. Switch cost of response time = response time of switch trials – response time of repeat trials; 100% denotes 100% valid cue; 75% denotes 75% valid cue; 50% denotes 50% valid cue; ^∗^*p* < 0.050; ^∗∗^*p* < 0.010.

The main behavioral variable was the switch cost of response time, which was defined as the difference in the reaction times between the switch and repeat trials. Two-way repeated measure ANOVA of validity (100, 75 versus 50%) × group (open-skilled, closed-skilled versus control) testing the effects on the switch cost of response times indicated that the validity × group effect on the switch cost of response times was marginally significant (*p* = 0.053). Participants in the open-skilled group showed significantly fewer switch cost values than the closed-skilled (*p* = 0.023) and control (*p* < 0.001) groups in the 100% validity condition (Figure 2). Participants in both the open- (*p* < 0.001) and closed-skilled (*p* = 0.033) groups showed significantly fewer switch cost values than the control group in the 50% validity condition (Figure 2). No significant differences in switch costs were revealed between the open- and closed-skilled groups (*p* = 0.473) (Figure 2). Their details can be found in Yu et al. (2017).

### Cue-Locked P3 (350–550 ms)

**FIGURE 3:**
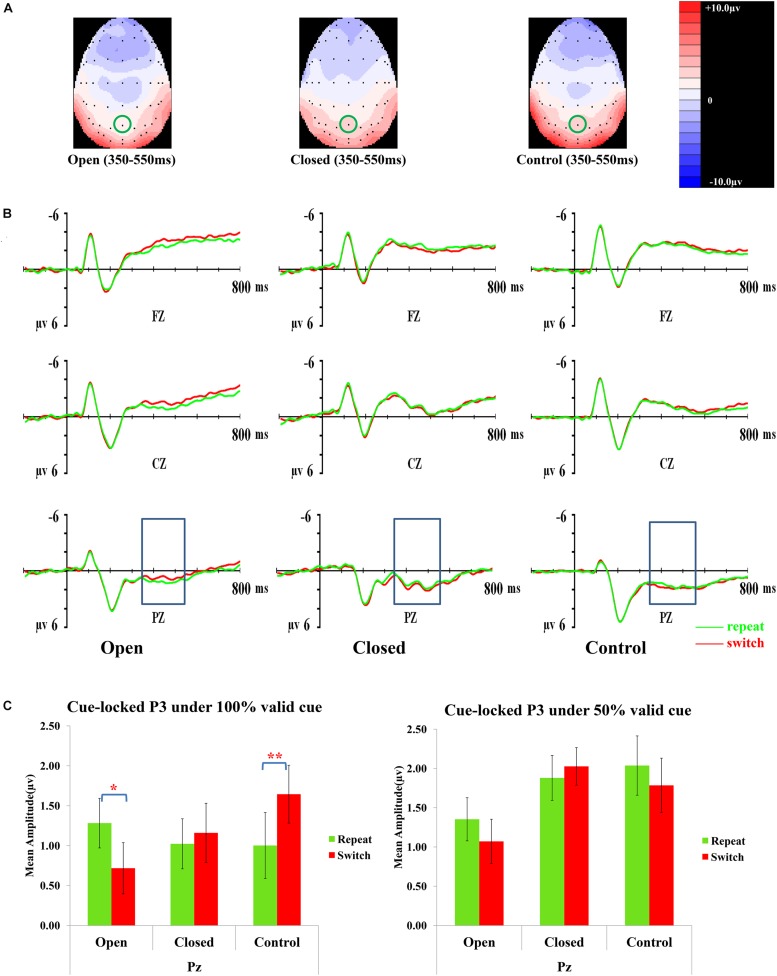
Analyses of the cue-locked P3 among three groups of participants. **(A)** Topographical distributions within the 350–550 ms post-cue time-window regardless of trial types and validities; the Pz site is indicated with the green circle. **(B)** Mean amplitudes of the waveforms of the repeat (in green) and switch (in red) trial-types regardless of validities; the 350–550 ms time-window at Pz is indicated with the rectangular boxes. **(C)** Comparisons of the mean amplitudes between the repeat and switch trial-types at the Pz site for the 100% (left panel) and 50% (right panel) validity conditions. Error bars indicate SEM; ^∗^*p* < 0.050; ^∗∗^*p* < 0.010.

Figure 3 presents topographic maps (3A) and waveforms (3B) of the cue-locked P3 for the open-skilled, closed-skilled, and control groups. The covariate of years of professional motor skill practices [*F*(1,50) = 0.54, *p* = 0.466, ${\text{η}}_{\text{p}}^{2}$ = 0.011] was not significant. The validity × trial × site × group effect [*F*(6.264,156.602) = 2.66, *p* = 0.016, ${\text{η}}_{\text{p}}^{2}$ = 0.096] was found significant. The site main effect was also significant [*F*(1.488,74.389) = 9.61, *p* = 0.001, ${\text{η}}_{\text{p}}^{2}$ = 0.161]. However, the validity [*F*(2,100) = 0.13, *p* = 0.880, ${\text{η}}_{\text{p}}^{2}$ = 0.003], trial [*F*(1,50) = 0.002, *p* = 0.968, ${\text{η}}_{\text{p}}^{2}$ < 0.001], and group effects [*F*(2,50) = 0.42, *p* = 0.657, ${\text{η}}_{\text{p}}^{2}$ = 0.017] on the amplitudes of cue-locked P3 were not significant.

*Post hoc* analyses on trial × site × group effect were conducted separately at each level of validity. The trial × site × group effect was only significant in the 100% validity condition [*F*(3.351,85.442) = 5.46, *p* = 0.001, ${\text{η}}_{\text{p}}^{2}$ = 0.177] but not in the 75% [*F*(2.752,70.179) = 1.11, *p* = 0.348, ${\text{η}}_{\text{p}}^{2}$ = 0.042] and 50% validity conditions [*F*(3.249,82.856) = 0.13, *p* = 0.952, ${\text{η}}_{\text{p}}^{2}$ = 0.005]. Hence, the trial × group effect for the 100% validity condition was further examined at each of the electrode sites. The trial × group effect was significant at Fz [*F*(2,51) = 6.82, *p* = 0.002, ${\text{η}}_{\text{p}}^{2}$ = 0.211] and Pz [*F*(2,51) = 8.03, *p* = 0.001, ${\text{η}}_{\text{p}}^{2}$ = 0.239] but not at Cz [*F*(2,51) = 1.32, *p* = 0.28, ${\text{η}}_{\text{p}}^{2}$ = 0.049]. At Fz, the open-skilled group showed marginally less positive-going cue-locked P3 in the switch than repeat trials (*p* = 0.056) in the 100% validity condition, whereas the closed-skilled group showed an opposite trend whereby the cue-locked P3 was significantly more positive-going in the switch than repeat trials (*p* = 0.003). The control group did not show significant between-trial-type differences in the amplitudes of cue-locked P3 at Fz (*p* = 0.822) (Figure 3C). At Pz, the open-skilled group showed a less positive-going cue-locked P3 in the switch than repeat trials (*p* = 0.011). The closed-skilled group, however, did not show significant between-trial-type differences in the amplitudes of cue-locked P3 at Pz (*p* = 0.523). The control group showed significantly more positive-going cue-locked P3 at Pz in the switch than repeat trials (*p* = 0.004).

### CNV (1200–1500 ms)

**FIGURE 4:**
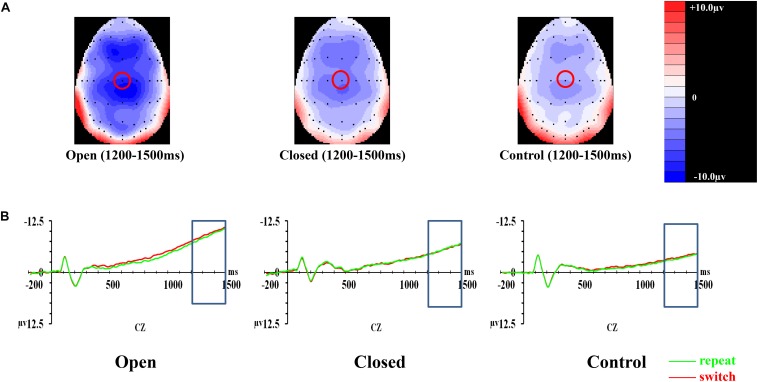
Analyses of CNV among three groups of participants. **(A)** Topographical distributions within the 1000–1500 ms post-cue time-window regardless of trial types and validities; the Cz site is indicated with the red circle. **(B)** Mean amplitudes of the waveforms of the repeat (in green) and switch (in red) trial-types extracted at the Cz site regardless of validities; the 1000–1500 ms time-window is indicated with the rectangular boxes.

In CNV, the covariate of years of professional motor skill practices was found significant [*F*(1,50) = 4.21, *p* = 0.045, ${\text{η}}_{\text{p}}^{2}$ = 0.078]. The validity × trial × site × group effect [*F*(6.118,152.946) = 0.55, *p* = 0.772, ${\text{η}}_{\text{p}}^{2}$ = 0.022] on the amplitudes of CNV was not significant (Figure 4). The validity effect [*F*(2,100) = 7.20, *p* = 0.001, ${\text{η}}_{\text{p}}^{2}$ = 0.126] was significant. However, trial [*F*(1,50) = 0.06, *p* = 0.811, ${\text{η}}_{\text{p}}^{2}$ = 0.001], site [*F*(2,100) = 1.54, *p* = 0.221, ${\text{η}}_{\text{p}}^{2}$ = 0.030], and group effects [*F*(2,50) = 1.06, *p* = 0.354, ${\text{η}}_{\text{p}}^{2}$ = 0.041] were not significant. *Post hoc* analysis showed that CNV in the 100% validity condition was more negative-going than those in 75% (*p* < 0.001) and 50% (*p* < 0.001) validity conditions.

### N2 (200–300 ms)

**FIGURE 5:**
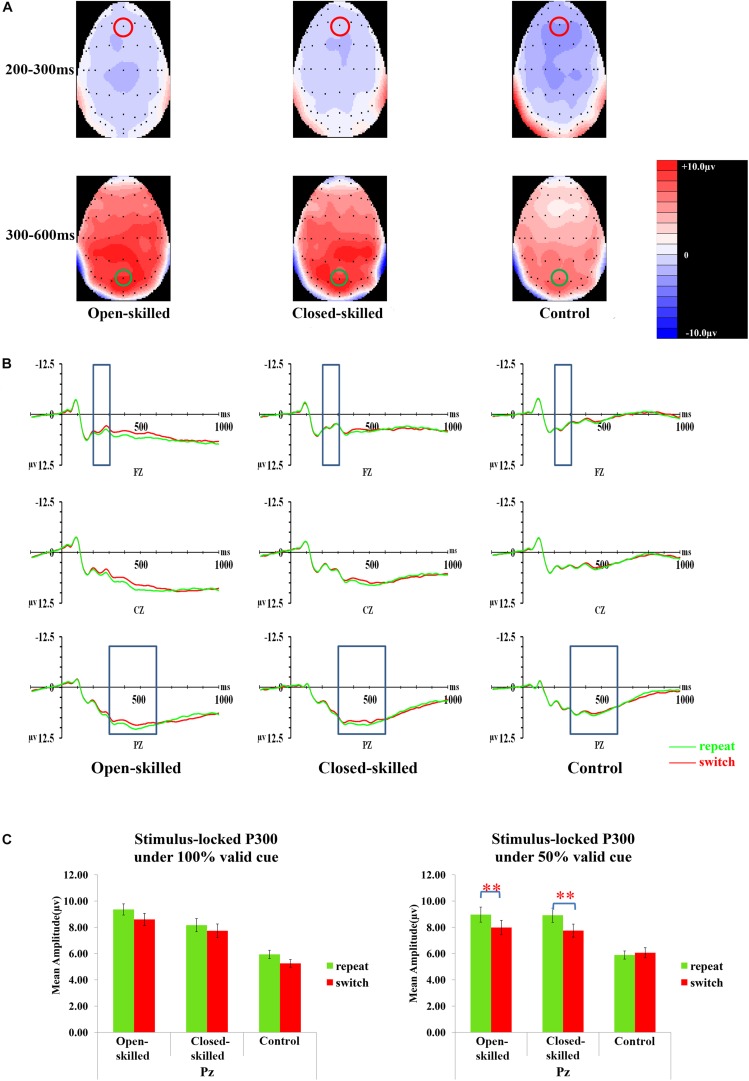
Analyses of the N2 and stimulus-locked P3 among three groups of participants. **(A)** Topographical distributions of N2 (200–300 ms) and stimulus-locked P3 (300–600 ms) post-target time-window regardless of trial types and validities; the Fz site is indicated with the red circle, and the Pz site is indicated with the green circle. **(B)** Mean amplitudes of the waveforms of the repeat (in green) and switch (in red) trial-types regardless of validities; the 200–300 ms time-window at Fz and 300–600 ms time-window at Pz are indicated with the rectangular boxes. **(C)** Comparisons of the mean amplitudes between the repeat and switch trial-types at the Pz site for the 100% (left panel) and 50% (right panel) validity conditions. ^∗∗^*p* < 0.010.

The covariate of years of professional motor skill practices was found not statistically significant [*F*(1,50) = 0.29, *p* = 0.596, ${\text{η}}_{\text{p}}^{2}$ = 0.006]. The validity [*F*(1.656,82.825) = 1.85, *p* = 0.170, ${\text{η}}_{\text{p}}^{2}$ = 0.036], trial [*F*(1,50) = 1.63, *p* = 0.208, ${\text{η}}_{\text{p}}^{2}$ = 0.031], group [*F*(2,50) = 0.42, *p* = 0.658, ${\text{η}}_{\text{p}}^{2}$ = 0.017], and the validity × trial × site × group effects [*F*(5.866,146.655) = 1.23, *p* = 0.295, ${\text{η}}_{\text{p}}^{2}$ = 0.047] on the amplitudes of N2 were also not significant. However, the site [*F*(1.275,63.730) = 5.86, *p* = 0.012, ${\text{η}}_{\text{p}}^{2}$ = 0.105], and validity × trial × site × covariate (years of professional motor skill practices) effect were significant [*F*(2.933,146.655) = 2.80, *p* = 0.043, ${\text{η}}_{\text{p}}^{2}$ = 0.053]. *Post hoc* analysis showed that N2 in 100% validity conditions was more negative-going in the switch than repeat trials at Fz (*p* = 0.034), Cz (*p* < 0.01), and Pz (*p* < 0.01), but this effect was not found in 75 and 50% validity conditions. In both 75 and 50% validity conditions, N2 was more negative-going at Fz (*ps* < 0.05) than Cz and Pz. Figure 5A presents the topographic maps of the N2 component.

### Stimulus-Locked P3 (300–600 ms)

The topographic maps (5A) and waveforms (5B) of the stimulus-locked P3 for the open-skilled, closed-skilled, and control groups are presented in Figure 5. The covariate of years of professional motor skill practices was significant [*F*(1,50) = 4.46, *p* = 0.040, ${\text{η}}_{\text{p}}^{2}$ = 0.082]. The validity × trial × site × group effect [*F*(5.671,141.771) = 2.25, *p* = 0.045, ${\text{η}}_{\text{p}}^{2}$ = 0.082] on the amplitudes of stimulus-locked P3 was found significant. The site [*F*(1.474,73.719) = 4.14, *p* = 0.031, ${\text{η}}_{\text{p}}^{2}$ = 0.076] and group effects [*F*(2,50) = 5.27, *p* = 0.008, ${\text{η}}_{\text{p}}^{2}$ = 0.174] were significant. The trial effect was marginally significant [*F*(1,50) = 3.93, *p* = 0.053, ${\text{η}}_{\text{p}}^{2}$ = 0.073]. However, the validity [*F*(2,100) = 0.33, *p* = 0.719, ${\text{η}}_{\text{p}}^{2}$ = 0.007] effect was not significant.

By adjusting years of professional motor skill practices, *post hoc* analysis showed significant trial × site × group effect only in the 50% validity condition [*F*(3.267,81.670) = 3.48, *p* = 0.017, ${\text{η}}_{\text{p}}^{2}$ = 0.122] (Figure 5C), but not in the 100% [*F*(2.824,70.610) = 1.81, *p* = 0.322, ${\text{η}}_{\text{p}}^{2}$ = 0.045] and 75% validity conditions [*F*(2.897,72.437) = 1.37, *p* = 0.260, ${\text{η}}_{\text{p}}^{2}$ = 0.052]. Interestingly, the results were different from those found in cue-locked P3, in which the same three-way interaction effect was significant in the 100% validity condition. The trial × group effect in the 50% validity condition was further tested separately for Fz, Cz, and Pz. The trial × group effect was found significant at the Cz and Pz [*F*(2,51) = 3.52, *p* = 0.037, ${\text{η}}_{\text{p}}^{2}$ = 0.121; *F*(2,51) = 4.14, *p* = 0.021, ${\text{η}}_{\text{p}}^{2}$ = 0.140, respectively], but not at the Fz [*F*(2,51) = 2.02, *p* = 0.143, ${\text{η}}_{\text{p}}^{2}$ = 0.073]. At Pz, both the open-skilled (*p* = 0.008) and closed-skilled (*p* = 0.002) groups showed significantly less positive-going stimulus-locked P3 in the switch than repeat trials (Figure 5C). The control group, however, showed no significant between-trial-type difference in the stimulus-locked P3 amplitudes at Pz (*p* = 0.630). At Cz, the open-skilled (*p* < 0.001) and closed-skilled (*p* = 0.038) groups had significantly less positive-going stimulus-locked P3 in the switch than repeat trials; whereas the control group did not show any significant between-trial-type differences in the amplitudes of stimulus-locked P3 (*p* = 0.838).

### Hierarchical Stepwise Regression

**FIGURE 6:**
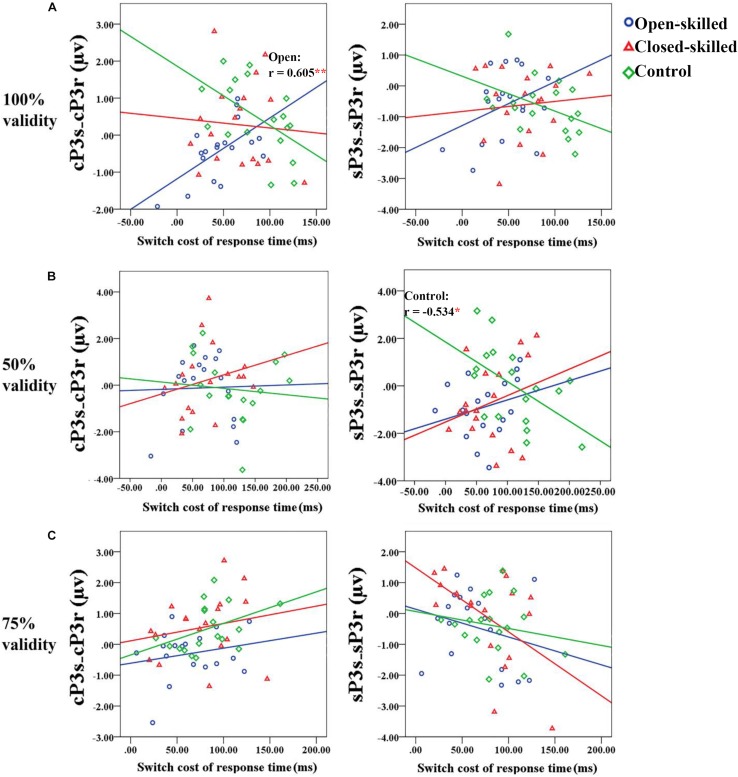
Scatter plots showing the relationships between the differences of the mean amplitudes of the cue-locked P3 between switch and repeat trial-types (cP3_S_-cP3_R_) (right panel) or that of the stimulus-locked P3 (sP3_S_-sP3_R_) (right panel) and the switch cost of response time for the 100, 50, and 75% validity conditions (from a to c) among the three groups of participants. **(A)** In the 100% validity condition, one significant correlation coefficient (*r* = 0.605, *p* = 0.008) is revealed in the open-skilled group for the cue-locked P3 (cP3s-cP3r); the blue regression line presents the significant positive correlation between the two variables. **(B)** In the 50% validity condition, one significant correlation coefficient (*r* = –0.534, *p* = 0.022) is revealed in the control group for the stimulus-locked P3 (sP3s-sP3r); the green regression line presents the significant negative correlation between the two variables. Only control group shows significant correlation. **(C)** In the 75% validity condition, no significant correlations are revealed. ^∗^*p* < 0.050; ^∗∗^*p* < 0.010.

In the 100% validity condition, the regression model was significant, *R*^2^ = 0.226, *F*(4,49) = 3.579, *p* = 0.012, with the only significant regressor in the model being the open-skilled group as a group identity (β = −0.573, *p* = 0.001) (Table 2). Other regressors (e.g., cP3_S_-cP3_R_ and sP3_S_-sP3_R_, closed-skilled group) were not significant (| β| < 0.285, *ps* > 0.057). The changes in the variance explained by the open-skilled group regressor were also significant [Δ*R*^2^ = 0.182, *F*(4,45) = 3.455, *p* = 0.015]. The effect of cP3_S_-cP3_R_ × open-skilled was significant (β = 0.475, *p* = 0.007), whereas cP3_S_-cP3_R_ (β = −0.431, *p* = 0.072) and cP3_S_-cP3_R_ × closed-skilled (β = 0.247, *p* = 0.205) did not show significant impacts (Figure 6A). These results suggested that the cP3_S_-cP3_R_, which was associated with proactive control for task switching, showed significant correlation with the switch cost of response times among the open-skilled participants but not among the closed-skilled and control participants.

**TABLE 2:** Results of hierarchical stepwise regression of amplitudes of the cue- and stimulus-locked P3 for predicting the switch cost of response times in the 100, 50, and 75% cue validity conditions.

	**100% cue validity**	**50% cue validity**	**75% cue validity**
	**Step 1**	**Step 1**	**Step 1**

	**Standardized regression coefficients (β)**	**Standardized regression coefficients (β)**	**Standardized regression coefficients (β)**

cP3_S_-cP3_R_	–0.075	–0.008	0.320^∗^
sP3_S_-sP3_R_	0.029	–0.062	–0.378^∗∗^
Open-skilled group	–0.573^∗∗^	−0.474^∗^	–0.139
Closed-skilled group	–0.284	–0.397^∗∗^	–0.051
R^2^	0.226	0.185	0.255
Adjusted R^2^	0.163	0.119	0.195
*F*	3.579^∗^	2.782^∗^	4.203^∗∗^

	**Step2**	**Step2**	**Step2**

	**Standardized regression coefficients (β)**	**Standardized regression coefficients (β)**	**Standardized regression coefficients (β)**

cP3_S_-cP3_R_	–0.431	0.010	0.445
sP3_S_-sP3_R_	–0.262	−0.550^∗^	–0.138
Open-skilled group	−0.393^∗^	−0.363^∗^	–0.133
Closed-skilled group	–0.265	−0.347^∗^	–0.057
cP3_S_-cP3_R_ × Open-skilled	0.475^∗∗^	0.066	–0.070
cP3_S_-cP3_R_ × Closed-skilled	0.247	0.131	–0.099
sP3_S_-sP3_R_ × open-skilled	0.293	0.464^∗^	–0.096
sP3_S_-sP3_R_ × closed-skilled	0.225	0.519^∗^	–0.255
R^2^	0.408	0.342	0.277
Adjusted R^2^	0.303	0.225	0.148
*F*	3.876^∗∗^	2.925^∗∗^	2.151
ΔR^2^	0.182	0.157	0.021
Δ*F*	3.455^∗^	2.685^∗^	0.329

*^∗^*p* < 0.050; ^∗∗^*p* < 0.010; RT denotes response time; cP3_S_-cP3_R_ denotes difference of mean amplitudes between switch and repeat trial-types of cue-locked P3; sP3_S_-sP3_R_ denotes difference of mean amplitudes between switch and repeat trial-types of stimulus-locked P3; 100% denotes 100% valid cue; 75% denotes 75% valid cue; 50% denotes 50% valid cue.*

In the 50% validity condition, the regression model was significant, *R*^2^ = 0.211, *F*(4,49) = 2.782, *p* = 0.037, for both the open- and closed-skilled groups as group identities were identified as significant regressors (β = −0.474, *p* = 0.003; β = −0.397, *p* = 0.012, respectively) (Figure 6B). The other two regressors, cP3_S_-cP3_R_ and sP3_S_-sP3_R_, were not significant (β = −0.008, *p* = 0.949; β = −0.062, *p* = 0.643, respectively). The changes in the variance are explained by the significance of the open- and closed-skilled groups’ regressors [Δ*R*^2^ = 0.157, *F*(4,45) = 3.057, *p* = 0.043]. The effects ofsP3_S_-sP3_R_ (β = −0.550, *p* = 0.020), open-skilled (β = −0.363, *p* = 0.031), closed-skilled (β = −0.347, *p* = 0.023), sP3_S_-sP3_R_ × open-skilled (β = 0.464, *p* = 0.022), andsP3_S_-sP3_R_ × closed-skilled (β = 0.519, *p* = 0.012) were the significant predictors, suggesting that the correlations between sP3_S_-sP3_R_ and the switch cost of response time in the open- and closed-skilled groups were significantly different from those of the control group. A follow-up analysis suggested that such correlation was significantly negative in the control participants (*r* = −0.534, *p* = 0.022), but not significant in the open-skilled (*r* = 0.262, *p* = 0.294) and closed-skilled (*r* = 0.274, *p* = 0.270) participants (Figure 6B).

In the 75% validity condition, the regression model was also significant, *R*^2^ = 0.255, *F*(4,49) = 4.203, *p* = 0.005, with cP3_S_-cP3_R_ (β = 0.320, *p* = 0.023) and sP3_S_-sP3_R_ (β = −0.378, *p* = 0.004) being significant regressors in the model. No other significant regressors were found [*R*^2^ = 0.277, *F*(8,45) = 2.151, *p* = 0.050] (Figure 6C). Both cP3s-cP3r (*r* = 0.317, *p* = 0.020) and sSP3s-sP3r (*r* = −0.333, *p* = 0.014) showed significant correlations with the participants’ switch cost of response times, regardless of the subgroups.

## Discussion

The current study investigated how motor skill experiences modulated proactive and reactive controls of executive function in healthy adults. New findings are that the open-skilled participants, when compared with the other two groups, showed significantly less positive-going parietal cue-locked P3 in switch than repeat trials, which coupled with better performances on task-switching in the predictive condition. These suggest that proactive control was unique to the open-skilled participants. It appears that they might have been able to deploy fewer attentional resources in proactively updating the new action rule than the closed-skilled participants. These findings corroborate with the results of the regression analysis, which show that better proactive control for task switching was associated with the between-trial difference in the cue-locked P3 amplitudes for the open- but not closed-skilled participants. On the contrary, in the non-predictive condition both the open- and closed-skilled participants showed significantly less positive-going parietal stimulus-locked P3 in the switch than repeat trials, which could not be found in the control participants. These results indicate that, prior experiences in motor skill training, regardless of the types of motor skills developed, would result in fewer deployments of attentional resources for reactively updating the new action rule under non-anticipatory circumstances. Our findings further confirm the dissociation of proactive and reactive controls in relation to their modulations by different motor-skill experiences. In particular, both proactive and reactive controls of executive functions could be enhanced by intensively exposing individuals to anticipatory and non-anticipatory enriched environments.

The results of the 75% validity condition will not be discussed because no significant findings were revealed in the comparisons of the ERP data.

### Proactive Control and Open-Skill Experience

The experimental task used in the present study required the participants to switch between two sets of action rules. Our ERP results show less positive cue-locked P3 in switch than repeat trials observed among the open-skilled participants, which was not the case in the closed-skilled and control groups. These findings indicate that open-skilled participants deployed fewer attentional resources when proactively updating the new action rule than their closed-skilled counterparts. In addition, behavioral results on the same groups of participants reported by in Yu et al. (2017) showed that open-skilled participants exhibited smaller switch cost of response times in the predictive condition (100% validity) than closed-skilled participants. The ERP findings of the cue-locked P3 and the published behavioral data suggest higher efficiency in updating the new action rule in proactive control than the closed-skilled participants. Our results are consistent with those reported in previous studies, which found participants engaged in open-skilled physical activities have higher efficiency in the updating process related to motor preparation. Open-skilled participants were proposed to have better motor-reprograming processes in terms of smaller timing errors than closed-skilled participants (Nakamoto and Mori, 2012). Jin et al. (2011) also found that professional badminton players had more accurate judgments of the placement of badminton strokes and larger P3a amplitude in the proactive anticipation than non-professional players, suggesting good anticipation ability. Mcrobert et al. (2011) also reported the open-skilled participants had the higher online updating ability, with which they were more adaptive to dynamic environments in the anticipation. Bertollo et al. (2016) further explained that the higher efficiency in switching between the automatic and controlled processes among sport experts than amateurs is a reason for the former to proficiently adapt to the changing environment in open sports. When compared with amateur athletes, the high efficiency found among the open-skilled athletes was revealed to result in the employment of less cognitive resources in controlled processes (Babiloni et al., 2010).

In the present study, the open-skilled participants are badminton athletes whose experience is in updating opponent’s changed kinematics information and overcoming interferences from previous deceptive movement patterns (Müller and Abernethy, 2012). They succeeded in winning the game mostly depending on how well they update environmental changes and anticipate their opponent’s actions (Bianco et al., 2017b). With such a background, the open-skilled participants tend to deploy fewer attentional resources when updating the new action rules in proactive control for the switch trials, which was not the case in the closed-skilled and control participants. The significant positive correlations between the amplitudes of cue-locked P3 and the switch cost of response time in our results were revealed only in the participants of the open-skilled group, which further substantiate the uniqueness of proactive control to the open-skilled participants.

No differences were revealed in the between-trial and between-group comparisons for the CNV. The non-significant between-group difference, supported by those reported in other study (Bianco et al., 2017b), suggests that the level of the top–down attentional control preparing for task switching was comparable across the three groups of participants. No difference between switch and repeat trials indicates that motor preparation levels were not affected by switch and repeat conditions, which was consistent with the findings in Gajewski and Falkenstein (2015). These results further support the notion that the CNV appears to be not an important neural marker related to the executive functions in the proactive control process. The years of professional motor skill practices is a significant covariate for CNV component, which suggests that participants with professional motor skill practices, regardless of any type of motor skills, had more negative-going CNV than those without professional motor skill practices. These findings were in line with those reported by Bianco et al. (2017a, b). The tonic activity in motor preparation was related to speed control in pre-supplementary motor area (Bianco et al., 2017a). In the present study, the speed requirement in both open (badminton) and closed (most were runners) skills was high, which may contribute to no group differentiation in CNV amplitude.

### Reactive Control and Motor Skill Experiences

The open- and closed-skilled participants showed less positive-going parietal stimulus-locked P3 in the switch than repeat trials in the non-predictive condition. Less positivity in the switch than repeat trials could not be found in the control group, who reported having no habit of practicing any types of physical activities. The results indicate that fewer attentional resources would have been deployed by the participants in updating the action rule in reactive control for switching in both open- and closed-skilled groups than in the control group. No significant differences were shown between these two motor-skilled groups, however. The comparable performance in stimulus-locked P3 for the open- and closed-skilled groups was not in line with our hypothesis, but was partly consistent with the findings in Wang et al.’s (2017). The findings of Wang and colleagues study revealed that open- and closed-skilled participants had comparable frontal N2 component and theta power for reactive control in a flanker task. The open-skilled participants, however, showed greater theta phase coherence (0–500 ms, 4 Hz; 300–400 ms, 5 Hz) for incongruent trials compared to congruent trials, but this effect could not be found in the closed-skilled group. These findings suggested that the dissociation between open- and closed-skilled participants appears to be the stability level of neural process rather than the level of allocated cognitive resources. Compared with those engaged in closed motor skills, the open motor skills required the participants to give a response within a limited time (Jin et al., 2011). Thus, the superior performance in reactive control of open-skilled participants could be showed in the paradigm with short interval between the response and the next target stimulus, like 500 ms in Tsai and Wang’s (2015), but not long interval between the response and the next target stimulus, like 2500 ms in the present study.

The behavioral results reported in Yu et al. (2017) indicated that open- and closed-skilled participants had significantly smaller switch cost of response times than controls. The ERP findings reported in this study reveal that both open-and closed-skilled groups had higher efficiency in updating the new action rule in reactive control, reflected by less positive-going stimulus-locked P3. These findings were supported by those reported in the previous studies (Kamijo and Takeda, 2010; Zhang et al., 2015). Kamijo and Takeda (2010) reported that the participants with regular physical training showed less switch cost and less positive stimulus-locked P3 in the switch than repeat trials than the sedentary controls, suggesting better reactive control in task switching. One plausible reason to account for the enhanced reactive control among the participants could have been the inevitable gains in cardiorespiratory fitness due to the intensive training received by both open- and close-skilled groups (Scisco et al., 2008; Tsai and Wang, 2015). Zhang et al. (2015) indicated that experienced fencers deployed less cognitive effort in the reactive inhibition process (as reflected from the less positive-going P3 in Nogo condition) than their novice counterparts, which is consistent with the results revealed in this study. Nevertheless, Yamashiro et al. (2015) revealed more positive somatosensory Nogo-P3 in the open-skilled participants for reactive control in the Go/Nogo task than the closed-skilled participants. The inconsistent findings reported in Yamashiro et al. (2015) study were likely due to a lack of controlling the years of professional motor skill practices among the participants, which could confound the results in the reactive control condition. Another reason may be that the superior performance in baseball group resulting from the baseball specific training, which could not be generalized to the closed-skilled participants.

Significant correlations were revealed between the amplitudes of stimulus-locked P3 and the switch cost of response time only in the control group. The results are unexpected, as significant correlations were anticipated among the open- and closed-skilled participants. A plausible explanation for the non-significant findings could be due to the heterogeneity of the strategies employed by the open- or closed-skilled participants. To further test this proposition, a median-split method (Tamura et al., 2010) was applied to subdivide the open- and closed-skilled groups into higher and lower ability subgroups based on the participants’ performances on between-trial difference in the stimulus-locked P3 amplitudes (sP3_S_-sP3_R_). Significant correlations were found in the higher but not the lower open-skilled ability subgroups; whilst significant correlations but opposite in direction were found in both the higher and lower closed-skilled ability subgroups. The small sample size for each of the subgroups (*n* = 9) only allowed us to suspect within-group heterogeneity as a possibly confounding factor to the non-significant relationships between the ERP and behavioral results. Future studies should explore possible deployment of different strategies by the participants in the same open- or closed-skilled group and the differences in the neural processes associated with proactive or reactive controls.

Our negative-going frontal N2 findings in the switch than repeat trials (in the 100 validity) at the Fz, Cz, and Pz electrodes are consistent with those reported by Hsieh and Wu (2011). The results suggested the possible involvement of response-set switching and suppression of conflict response processes unique to the behavioral task used in this study (Nakamoto and Mori, 2008; Wang et al., 2017), as no significant group difference was revealed in the N2 among all the participant groups.

### Limitations

First, the proactive and reactive control processes were prescribed by the switching task employed in this study. The results may not be directly generalized to other executive functions, such as inhibition or self-regulation. Second, the sample sizes of the open- and closed-skilled participants of this study were relatively small, which could have lowered the power of the analyses. Readers should be cautious when interpreting the results. Third, it is unclear whether badminton and track and field can best represent open- and closed-skilled physical activities, respectively. Any generalization of the findings should be restricted to participants of the same type of physical activities and level of competence. Future studies may consider recruiting participants of other types of physical activities and levels of competence. Fourth, the participants in the control group were those who had not been engaged in professional or amateur sports. The levels of physical activity engaged by these participants were not controlled. The existing differences between the open/closed-skilled and control groups could have been confounded by the differences in other parameters such as the levels of physical fitness rather than the types of motor skills, in case that the majority of the participants in the control group had been leading a sedentary lifestyle. Future study should recruit individuals who have comparable levels of physical fitness and/or physical training, but not at the professional level, as the control group. Fifth, the proposition of potential heterogeneity in strategies taken by the open- and closed-skilled groups was based on small sample sizes and without triangulation. Future studies should employ a more stringent research design and a larger sample size to address this issue.

## Conclusion

This study explored how experiences in open and closed motor skills modulate individuals’ proactive and reactive control processes. Compared with closed-skilled experiences, intensive open-skilled experiences were related to better proactive control for task switching characterized by lower switch cost and significant difference between switch and repeat trials of cue-locked P3 amplitudes. The enhanced proactive control is likely the result of high demand of anticipating environmental changes in open-skilled physical activities. Intensive open- and closed-skilled experiences were related to better reactive control for task switching than the experiences of control participants, which was most likely resulted from higher cardiorespiratory fitness. Proactive and reactive controls as part of the process of motor-skill development seem to be transferable to domain-general executive functions.

## Data Availability Statement

The datasets generated for this study are available on request to the corresponding author.

## Ethics Statement

The studies involving human participants were reviewed and approved by The Research Grant Committee of the Hong Kong Polytechnic University. The patients/participants provided their written informed consent to participate in this study.

## Author Contributions

AW and BL contributed to the data interpretation and article writing. BC contributed to the conceptualization, data interpretation, and article writing. CC contributed to the conceptualization, study design, data interpretation, and article writing. JP contributed to the data collection, data analysis, and data interpretation. QY contributed to conceptualization, study design, data collection, data analysis, data interpretation, and article writing.

## Conflict of Interest

The authors declare that the research was conducted in the absence of any commercial or financial relationships that could be construed as a potential conflict of interest.
